# Supplementary material for: SMYD3 protein degraders in HPV-negative head and neck squamous cell carcinoma
Source: bioRxiv. 2026 Sep 29:2026.09.28.754983. Preprint. [Version 1] doi: 10.64898/2026.09.28.754983 (PMC13648593; doi:10.64898/2026.09.28.754983)

bioRxiv preprint doi: https://doi.org/10.64898/2026.09.28.754983; this version posted September 29, 2026. The copyright holder for this preprint (which was not certified by peer review) is the author/funder. This article is a US Government work. It is not subject to copyright under 17 USC 105 and is also made available for use under a CC0 license.

Schema S1: Synthesis of common intermediate (S7)

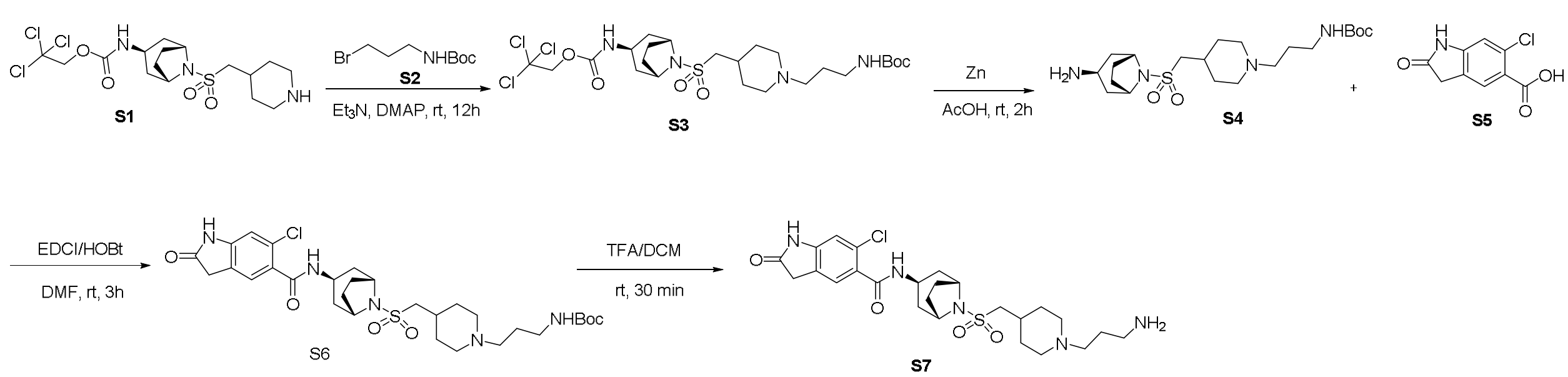

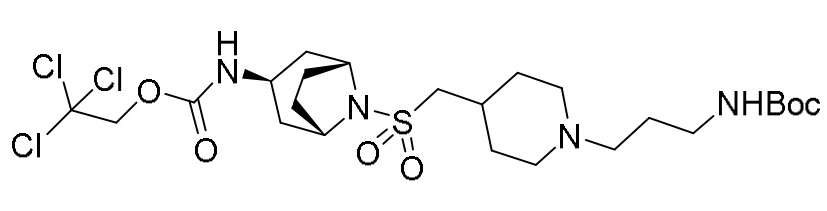

Supplement: Supplementary file 1 [file NIHPP2026.09.28.754983v1-supplement-15.pdf]
