# Supplementary material for: SMYD3 protein degraders in HPV-negative head and neck squamous cell carcinoma
Source: bioRxiv. 2026 Sep 29:2026.09.28.754983. Preprint. [Version 1] doi: 10.64898/2026.09.28.754983 (PMC13648593; doi:10.64898/2026.09.28.754983)
Supplement: Supplementary file 3 [file media-14.docx]

**Supplementary Table 7.** NMR spectra and UPLC-MS data of selected newly synthesized SMYD3 PROTAC compounds.

**^1^H NMR (500 MHz, DMSO-d6) spectrum of EPZ031686-IAP-01**

**UPLC-MS Data -EPZ031686-IAP-01**

**^1^H NMR (500 MHz, MeOD-d4) spectrum of EPZ031686-IAP-02**

**UPLC-MS Data -EPZ031686-IAP-02**

**^1^H NMR (500 MHz, MeOD-d4) spectrum of EPZ031686-IAP-03**

**UPLC-MS Data -EPZ031686-IAP-03**

**^1^H NMR (500 MHz, MeOD-d4) spectrum of EPZ031686-IAP-04**

**UPLC-MS Data -EPZ031686-IAP-04**

**^1^H NMR (400 MHz, MeOD-d4) spectrum of EPZ031686-IAP-05**

**UPLC-MS Data -EPZ031686-IAP-05**

**^1^H NMR (500 MHz, DMSO-d6) spectrum of EPZ031686-IAP-07**

**UPLC-MS Data -EPZ031686-IAP-07**

**H^1^ NMR (500 MHz, DMSO-d6) spectrum of EPZ031686-IAP-08**

**UPLC-MS Data -EPZ031686-IAP-08**

**H^1^ NMR (500 MHz, DMSO-d6) spectrum of EPZ031686-IAP-09**

**UPLC-MS Data -EPZ031686-IAP-09**

**^1^H NMR (500 MHz, MeOD-d4) spectrum of EPZ031686-IAP-10**

**UPLC-MS of EPZ031686-IAP-10**

**^1^H NMR (500 MHz, DMSO-d6) spectrum of EPZ031686-IAP-11**

**UPLC-MS of EPZ031686-IAP-11**

**^1^H NMR (500 MHz, MeOD-d6) spectrum of EPZ031686-CRBN-01**

**UPLC-MS of EPZ031686-CRBN-01**

**^1^H NMR (500 MHz, MeOD-d4) spectrum of EPZ031686-CRBN-02**

**UPLC-MS of EPZ031686-CRBN-02**

**^1^H NMR (500 MHz, DMSO-d6) spectrum of EPZ031686-CRBN-03**

**UPLC-MS of EPZ031686-CRBN-03**

**^1^H NMR (500 MHz, MeOD-d4) spectrum of EPZ031686-CRBN-04**

**UPLC-MS of EPZ031686-CRBN-04**

**^1^H NMR (500 MHz, DMSO-d6) spectrum of EPZ031686-CRBN-05**

**UPLC-MS of EPZ031686-CRBN-05**

**^1^H NMR (500 MHz, MeOD-d4) spectrum of EPZ031686-VHL-01**

**UPLC-MS of EPZ031686-VHL-01**

**^1^H NMR (500 MHz, MeOD-d4) spectrum of EPZ031686-VHL-02**

**UPLC-MS of EPZ031686-VHL-02**

**^1^H NMR (500 MHz, MeOD-d4) spectrum of EPZ031686-VHL-03**

**UPLC-MS of EPZ031686-VHL-03**

**^1^H NMR (500 MHz, MeOD-d4) spectrum of EPZ031686-VHL-04**

**UPLC-MS of EPZ031686-VHL-04**

**^1^H NMR (500 MHz, DMSO-d6) spectrum of EPZ -VHL-05**

**UPLC-MS of EPZ031686-VHL-05**
