# Supplementary material for: SMYD3 protein degraders in HPV-negative head and neck squamous cell carcinoma
Source: bioRxiv. 2026 Sep 29:2026.09.28.754983. Preprint. [Version 1] doi: 10.64898/2026.09.28.754983 (PMC13648593; doi:10.64898/2026.09.28.754983)
Supplement: Supplementary file 7 [file media-6.docx]

**Supplementary Table 1.** SMYD3 PROTAC structures with different E3 ligase ligands and bis-carboxyl linkers using EPZ031686 propyl amine as SMYD3 ligand.

| **Compound** | **SMYD3 ligand** | **Linker** | **E3 Ligase ligand** |
| --- | --- | --- | --- |
| IAP-01 |  |  |   IAP |
| IAP-02 |  |  |  |
| IAP-03 |  |  |  |
| IAP-04 |  |  |  |
| IAP-05 |  |  |  |
| IAP-07 |  |  |  |
| IAP-08 |  |  |  |
| IAP-09 |  |  |  |
| VHL-01 |  |  |   VHL |
| VHL-02 |  |  |  |
| VHL-03 |  |  |  |
| VHL-04 |  |  |  |
| CRBN-01 |  |  |   CRBN |
| CRBN-02 |  |  |  |
| CRBN-03 |  |  |  |
